# Integrated Bioinformatics Analysis Identifies a New Stemness Index-Related Survival Model for Prognostic Prediction in Lung Adenocarcinoma

**DOI:** 10.3389/fgene.2022.860268

**Published:** 2022-04-08

**Authors:** Shaohui Hou, Hongrui Xu, Shuzhong Liu, Bingjun Yang, Li Li, Hui Zhao, Chunyang Jiang

**Affiliations:** Department of Thoracic Surgery, Tianjin Union Medical Center, Nankai University, Tianjin, China

**Keywords:** LUAD, WGCNA, mRNAsi, survival, prognostic model

## Abstract

Background: Lung adenocarcinoma (LUAD) is one of the most lethal malignancies and is currently lacking in effective biomarkers to assist in diagnosis and therapy. The aim of this study is to investigate hub genes and develop a risk signature for predicting prognosis of LUAD patients. Methods: RNA-sequencing data and relevant clinical data were downloaded from The Cancer Genome Atlas (TCGA) and Gene Expression Omnibus (GEO) database. Weighted gene co-expression network analysis (WGCNA) was performed to identify hub genes associated with mRNA expression-based stemness indices (mRNAsi) in TCGA. We utilized LASSO Cox regression to assemble our predictive model. To validate our predictive model, me applied it to an external cohort. Results: mRNAsi index was significantly associated with the tissue type of LUAD, and high mRNAsi scores may have a protective influence on survival outcomes seen in LUAD patients. WGCNA indicated that the turquoise module was significantly correlated with the mRNAsi. We identified a 9-gene signature (CENPW, MCM2, STIL, RACGAP1, ASPM, KIF14, ANLN, CDCA8, and PLK1) from the turquoise module that could effectively identify a high-risk subset of these patients. Using the Kaplan-Meier survival curve, as well as the time-dependent receiver operating characteristic (tdROC) analysis, we determined that this gene signature had a strong predictive ability (AUC = 0.716). By combining the 9-gene signature with clinicopathological features, we were able to design a predictive nomogram. Finally, we additionally validated the 9-gene signature using two external cohorts from GEO and the model proved to be of high value. Conclusion: Our study shows that the 9-gene mRNAsi-related signature can predict the prognosis of LUAD patient and contribute to decisions in the treatment and prevention of LUAD patients.

## Introduction

Lung cancer causes over one million deaths every year and, as such, is considered a malignant disease associated with high mortality rates. Approximately 85% of all lung cancers are non-small cell lung cancer (NSCLC) and NSCLCs are considered the predominant histological type (Siegel et al., 2022). Within this histological type, lung adenocarcinoma (LUAD) is the most common subtype (Barlesi et al., 2016). While there have been significant improvements in multimodal therapy, such as those utilizing a combination of surgery, and targeted therapy. Patients diagnosed with LUAD still have notably poor outcomes, largely due to an absence of early diagnostic tools and predictive biomarkers (Brody, 2020).

There are still no definite conclusions about the origin of LUAD and its pathological mechanism. However, increased use of microarray profiles and genome-wide sequencing have recently been involved in the identification of important molecular prognostic factors allowing for more precise classification of LUAD and, thus, increasingly personalized treatment options can be made available to patients (Chang et al., 2020). Identifying molecular characteristics of LUAD may provide effective tools for predicting patient prognosis and LUAD’s response to therapy, thus improving physicians’ ability to individualize LUAD treatment (Langfelder and Horvath, 2008).

A variety of studies have indicated that tumor stem cells are not only valuable in research, but also are important in tumor differentiation, ultimate metastasis, and the development of drug resistance (Malta et al., 2018). The development of the mRNA expression-based stemness indices (mRNAsi) has allowed researchers to quantitatively assess stemness. By using mRNAsi to measure tumor development, scientists can evaluate the trustworthiness of stem cell indices for investigating tumors using data from TCGA (Pan et al., 2019). Expression data ranging from 0 to 1 is used to calculate the mRNAsi. Expression data with values closer to 1 indicate strong stem cell characteristics and low cell differentiation. Higher mRNAsi scores are correlated with increased biological activity in CSCs and more dedifferentiation of tumors, as shown by histopathological grading (Shibue and Weinberg, 2017).

Weighted gene co-expression network analysis (WGCNA) is a tool that systematic describes biological patterns seen in gene associations found between samples. WGCNA can be used to identify biomarker genes that may make good therapeutic targets by using information on the interconnectivity of gene sets and associations between different phenotypes and gene sets (Xu et al., 2020). WGCNA focuses on information from thousands of genes that are the most varied or all the genes, as opposed to only differentially expressed genes, to determine associations between genotypes and phenotypes and identify gene sets that may be of interest. In WGCNA, genes that have comparable expression patterns may also have similar regulatory networks and/or correlations in function or be members of the same pathway; and the gene network fits to a scale-free distribution model. The gene network can then be divided, based on these characteristics, into modules using qualifiers such as similar expression, resulting in the identification of hub genes (Zhang et al., 2020).

Considering the strong association between tumor stem cells and tumor pathogenesis, our study aimed to obtain a module that is closely related to stem cell characteristics and develop a new mRNAsi-related signature with hub genes in the module. Following development of the risk signature, its relationship with clinicopathological characteristics and prognosis in LUAD was investigated. An external validation was also conducted using GEO datasets to prove the predictive value of the risk signature.

## Materials and Methods

### Data Acquisition, Immune Score Generation, and Clinical Relationship

We collected gene expression profiling data from past LUAD patients that were included in the TCGA database so that 513 lung adenocarcinoma and 56 normal tissue samples were utilized in our study (https://portal.gdc.cancer.gov/). Corresponding patient data, such as age, gender, living status, tumor status, TNM stage, radio- and chemotherapy and survival data, and were available from TCGA. During data preprocessing we transformed gene names into official gene symbols using Perl language, and genes needed to have a non-zero expression value in a minimum of half of the sample type to be kept.

### Identification of Differentially Expressed Genes (DEGs)

Differentially expressed analysis was conducted by the package “limma”, and the cut-off criteria was set as |log (Fold Change)| > 1 and *p*-value < 0.05 (Pencina and D’agostino, 2004). The R package “pheatmap” was used to draw heatmap and volcano plot. The box plots of the key genes for validation were plotted by R, using the package “ggpubr”.

### Weighted Gene Co-Expression Network Analysis

We constructed co-expression modules using the WGCNA R package based off the DEGs we identified between normal and tumor tissues in LUAD patients. WGCNA was able to group highly correlated genes and identify important modules or genes that are associated with clinical characteristics of interest. We also used Pearson correlation coefficients between each identified gene module to construct a matrix than can establish module-trait relationships between DEG expression and the associated mRNAsi with respect to the β value (soft threshold value). After the most important module was identified we then calculated the gene significance (GS) and module membership (MM). We defined key genes as those with the GS > 0.7 and MM > 0.7 for this module.

### Function Enrichment Analysis (GO/KEGG)

We used Gene Ontology (GO) and Kyoto Encyclopedia of Genes and Genomes (KEGG) analysis with the “clusterProfiler” R package to explore potential gene pathways and biological functions in the significant module. Statistical significance was defined as a *P*. adjust value < 0.05. The R package “org.Hs.eg.db” was used to map important genes with the Ensemble ID. Bubble plots were assembled by R to visualize the top 10 results.

### PPI Network Construction

The protein-protein interaction (PPI) networks were constructed based on genes in the most mRNAsi-related module, using the STRING database (version 11.0) of known and predicted protein-protein interactions (Ritchie et al., 2015), which now covers 24,584,628 proteins from 5,090 organisms. The users only need to submit a list of gene symbols and species, and the website provides interaction relationships among submitted proteins. These interactions include direct (physical) and indirect (functional) associations.

### PCA Analysis and Subgroup Analysis

To study the function of hub genes in the key module, we separated 513 LUAD patients into different subgroups by the approach of “ConsensusClusterPlus”, an algorithm for determining clusters using an unsupervised analysis based on gene expression (Szklarczyk et al., 2019). The consensus clustering tool provides users with quantitative and qualitative evidence that allows the estimation of unsupervised class counts in a dataset. The maximum evaluated k (max K) was set to 9 and other parameters of ConsensusClusterPlus were set to default. The R package (R v3.5.1) of PCA analysis was adopted to explore the gene expression patterns in subgroups of LUAD.

### Construction and Validation of mRNAsi-Related Risk Signature

To explore the prognostic value of hub genes in hub module resulting from WGCNA, we used a least absolute shrinkage and selection operator (LASSO) regression to narrow the range of target genes via the “glmnet” R package because the predictor variable was much larger than the sample content in the gene expression data. The risk score formula for predicting the prognosis of LUAD patients was: risk score = the sum of the multivariate LASSO regression coefficient ratio of each mRNA multiplied by the expression level of each mRNA. We divided the LUAD patients into two groups, high- and low-risk, based on the median value of risk score. In the two groups, the clinicopathological characteristics of each patient, including age, gender, living status, tumor status, TNM stage, survival status, chemotherapy, radiotherapy, and gene expression profile, were presented via the “pheatmap” and “survival” R packages. In addition, we conducted time-dependent receiver operating characteristic (tdROC) curve and Kaplan-Meier survival curve analyses to validate the signature in both the training set and testing set. Log-rank test was applied to calculate the difference of overall survival rate between the high-risk and low-risk groups. “*p* < 0.05” was considered statistically significant. In the validation phase, we verified the nomogram in the GEO by using another LUAD cohort, GSE17536 and GSE17537.

### Exploration of Clinical Independence and Construction of the Nomogram

To validate the independence of the risk model, we conducted univariate and multivariate Cox regression analyses to evaluate the predictive efficacy of the model. A nomogram encompassing the risk score model and clinicopathological factors was plotted by the “rms” R package. The accuracy of the nomogram was examined using the consistency between the actual and the predicted outcomes. Next, we submitted these outcomes to the calibration curve to visualize the performance of the nomogram. The 45° line represented the best prediction (Wilkerson and Hayes, 2010). Based on the different clinicopathological characteristics and the risk score of each patient, we calculated the total score to predict 1, 3, and 5-years prognosis of LUAD patients. To determine the prognostic value of the nomogram, we use the Kaplan–Meier survival curve to compare the predictive value of nomogram for OS. The predictive efficiency of the nomogram for 1/3/5-years survival was assessed using the tdROC curve analysis.

### Statistical Analysis

Differences between variables were assessed with independent *t*-tests. Kaplan-Meier curves and log-rank tests were used to analyze the survival data, and univariate Cox regression analysis was used to identify independent prognostic factors. Time-dependent ROC analysis was used to evaluate the accuracy of the prognostic predictive model. The area under the ROC curve (AUC) > 0.60 was regarded as acceptable for predictions, and AUC >0.75 was deemed to have great predictive value. R software was used to perform all statistical analyses, and *p* < 0.05 was considered statistically significant.

## Results

### Weighted Co-Expression Network Construction and Significant Module Identification

**FIGURE 1:**
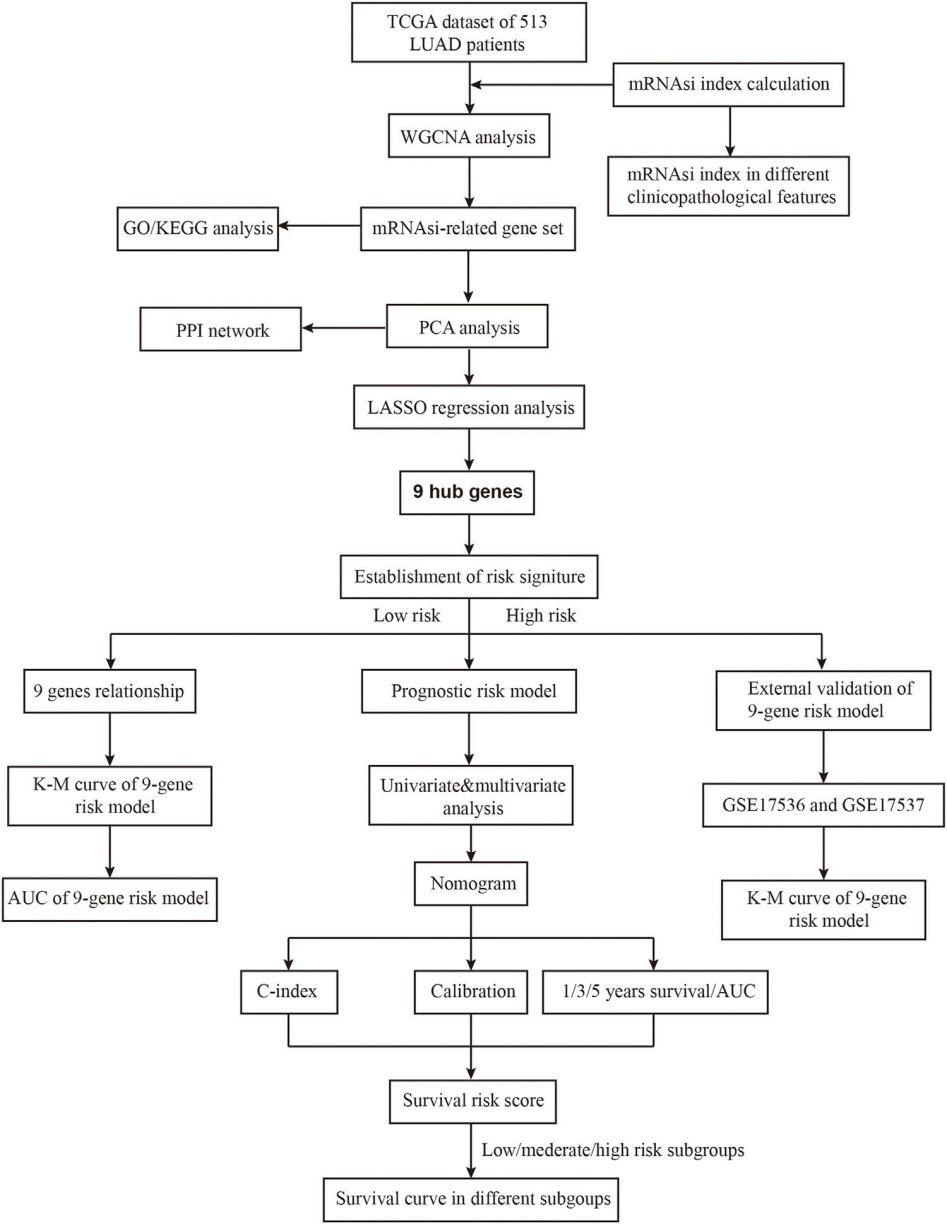
The flowchart of this study.

**FIGURE 2:**
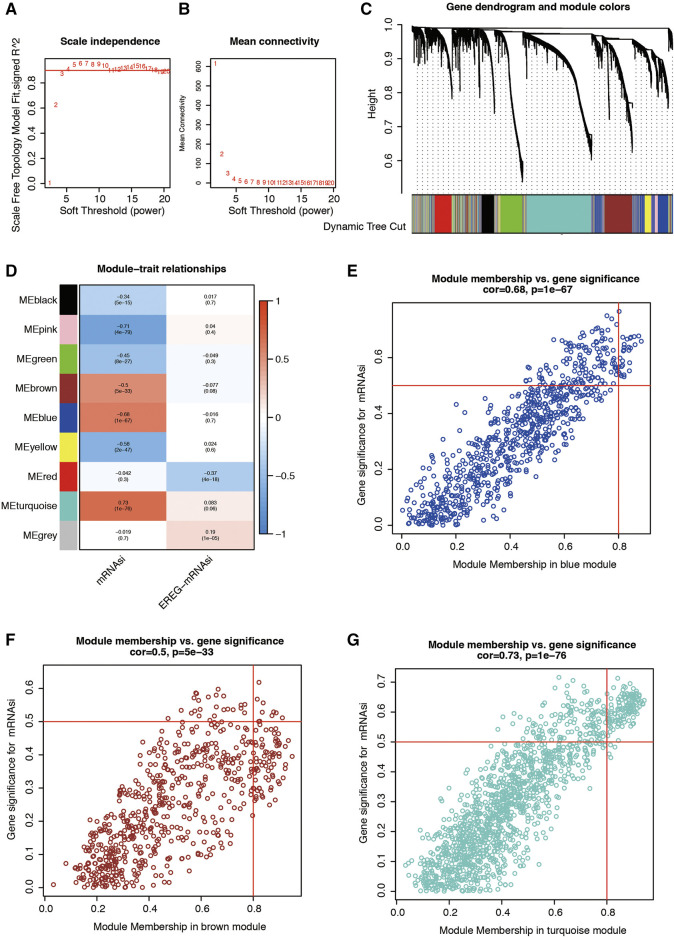
**(A,B)** Graphs of scale independence, mean connectivity and scale-free topology, the appropriate soft-power was 4. **(C)** Cluster dendrogram of the co-expression network modules (1-TOM). **(D)** Correlation between the gene module and clinical characteristics, including the mRNAsi and EREG-mRNAsi. **(E)** Scatter diagram for MM vs. GS for the mRNAsi in the blue module. **(F)** Scatter diagram for MM vs. GS for the mRNAsi in the brown module. **(G)** Scatter diagram for MM vs. GS for the mRNAsi in the turquoise module. LUAD, lung adenocarcinoma; mRNAsi, mRNA expression-based stemness index; EREG, epigenetically regulated.

The flowchart shown in Figure 1 summarizes the overall bioinformatics analysis of our study. The WGCNA R package was applied to build a co-expression network, and 4,430 differentially expressed genes (DEGs) was listed in Supplementary Table S1. In this study, we set the soft threshold to the power of β = 4 to guarantee a scale-free network (Figures 2A,B). To investigate the gene sets that are associated with mRNAsi of LUAD, we applied WGCNA because it defines transcriptional modules using Pearson correlation and establishes a relationship between different colored modules and clinical traits. As a result, we identified 9 distinct co-expression modules (Figure 2C).

To detect whether modules were significantly associated with specific clinical traits, we looked for strong associations between eigengenes and external traits. The correlation between module and trait was then visualized as a heatmap (Figure 2D). These results showed that patient disease state is significantly correlated with seven modules. Based on the correlation coefficients, genes clustered in black, pink, green, brown, blue, and yellow modules are downregulated in LUAD tissues, while genes in the turquoise module are highly expressed in LUAD tissues. Genes clustered in turquoise modules have the strongest positive correlation (Cor = 0.7, *p* = 1e-76) with patients’ disease status. This data indicates that genes in the turquoise module are significantly associated with the mRNAsi of LUAD patients. The scatterplot below illustrates the strength of the link between the mRNAsi signature and the module membership for each gene found in blue, brown, and turquoise modules (Figures 2E–G).

### Functional Enrichment Analyses of Genes in Turquoise Co-expression Modules

**FIGURE 3:**
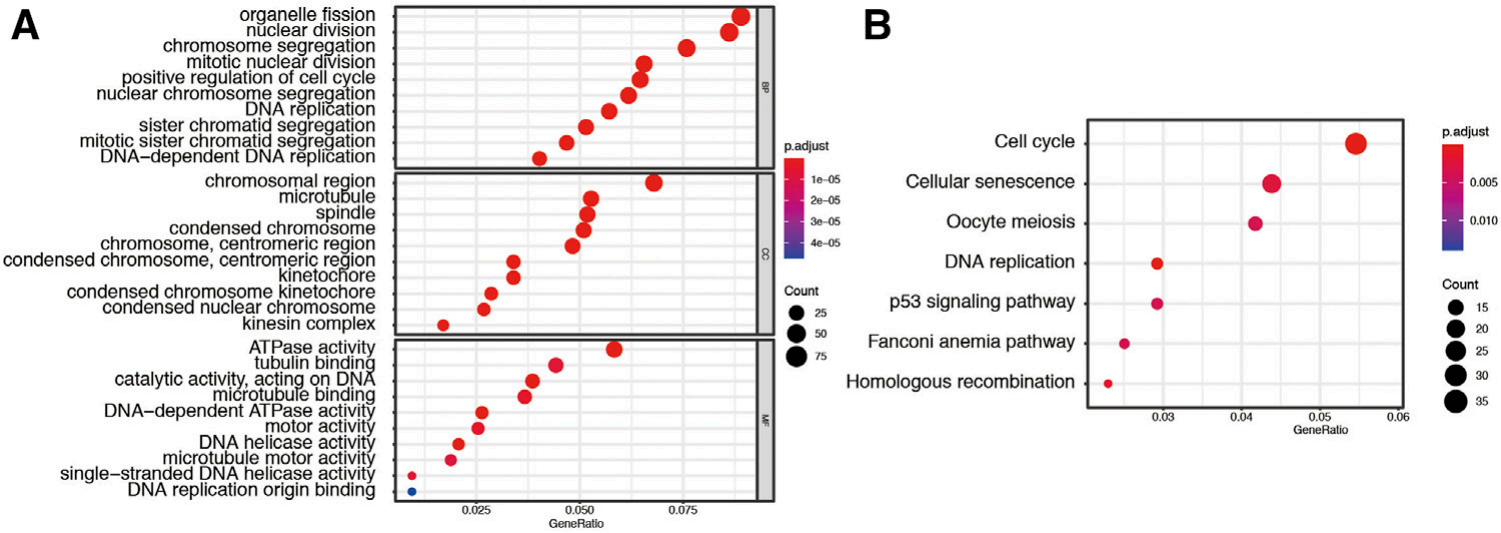
Functional analysis for genes in turquoise module. **(A)** Representative results of GO analysis. **(B)** Representative results of KEGG analysis.

Next, the turquoise module underwent GO and KEGG pathway analysis and we identified the top 10 GO and top 7 KEGG pathway enrichment terms, as shown in Figures 3A,B. The GO analysis showed that this module is enriched in organelle fission, chromosomal, and ATPase activity. The KEGG analysis indicated that the genes are mainly involved in cell cycle, cellular senescence, oocyte meiosis et al., which are pivotal in the regulation of immune responses.

### Correlation of the mRNAsi, Clinical Characteristics, and Key Genes in the Turquoise Module

**FIGURE 4:**
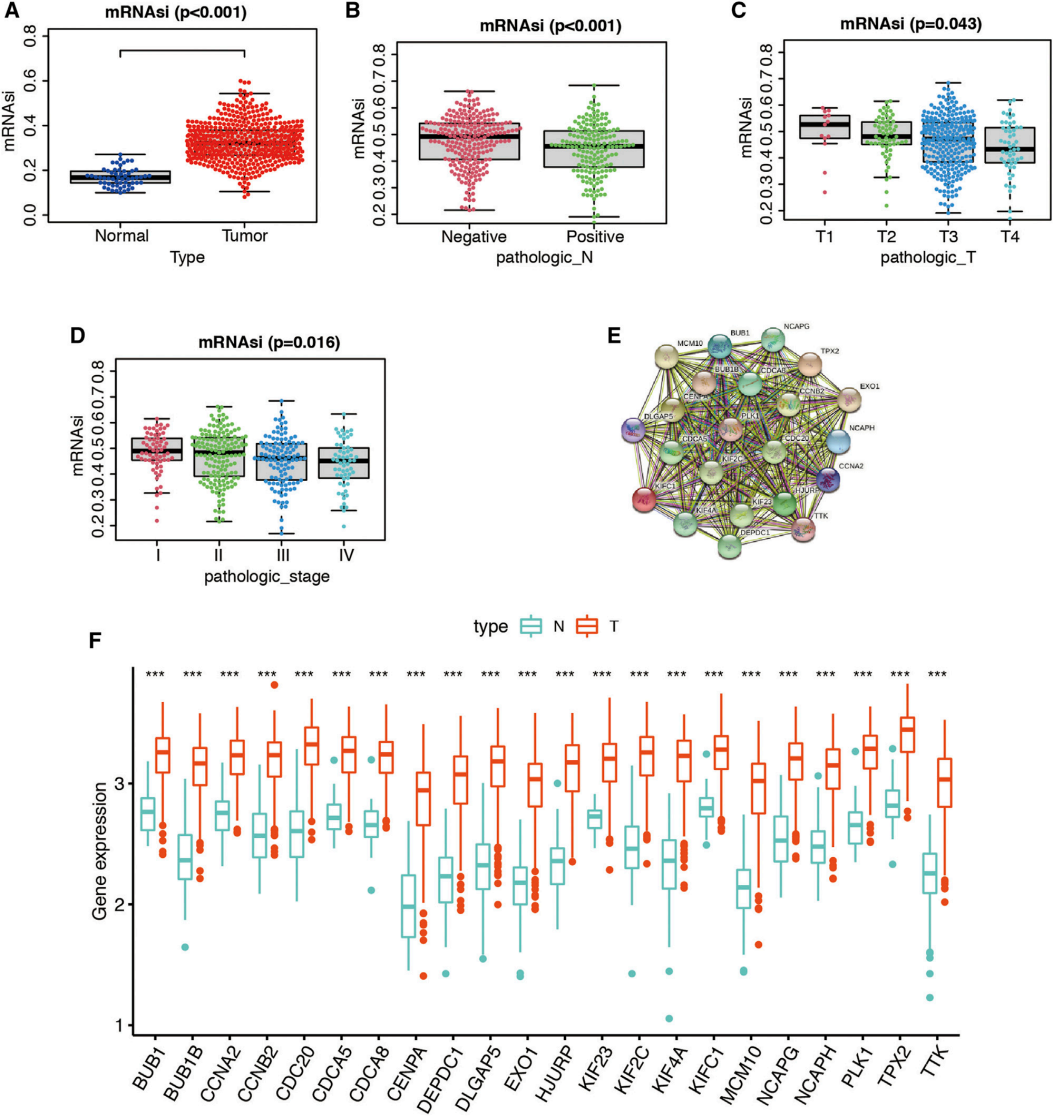
mRNAsi differences in different clinicopathological features. **(A)** Differences in the mRNAsi between normal and LUAD tissues. **(B)** Differences in the mRNAsi between different lymph node metastasis. **(C)** Differences in the mRNAsi among different T stages. **(D)** Comparison of the mRNAsi among four AJCC stages. **(E)** Protein–protein interactions between 22 hub genes in turquoise module. The thickness of the solid line represents the strength of the relationship. **(F)** Differential expression of 22 hub genes between normal and tumor cases in the LUAD dataset of TCGA shown by box-plots.

As indicated in Figure 4A, we found a notable difference between the mRNAsi of LUAD and the mRNAsi of normal tissues. The mRNAsi of LUAD tissues was found to be higher compared to that of normal tissues. We also observed significant differences in N stage (Figure 4B), T stage (Figure 4C), and AJCC stage (Figure 4D). The PPI network, consisting of the top 22 hub genes in turquoise module, was constructed using the STRING database. In total, 22 nodes and 460 edges were included in this PPI network (Figure 4E), with an average node degree of 20.9 and strong correlations. The expression levels of the top 22 hub genes were higher in tumor tissue than in normal tissue (Figure 4F).

### Consensus Clustering of Genes in the Turquoise Module and Identification of Two Clusters of LUAD Patients

**FIGURE 5:**
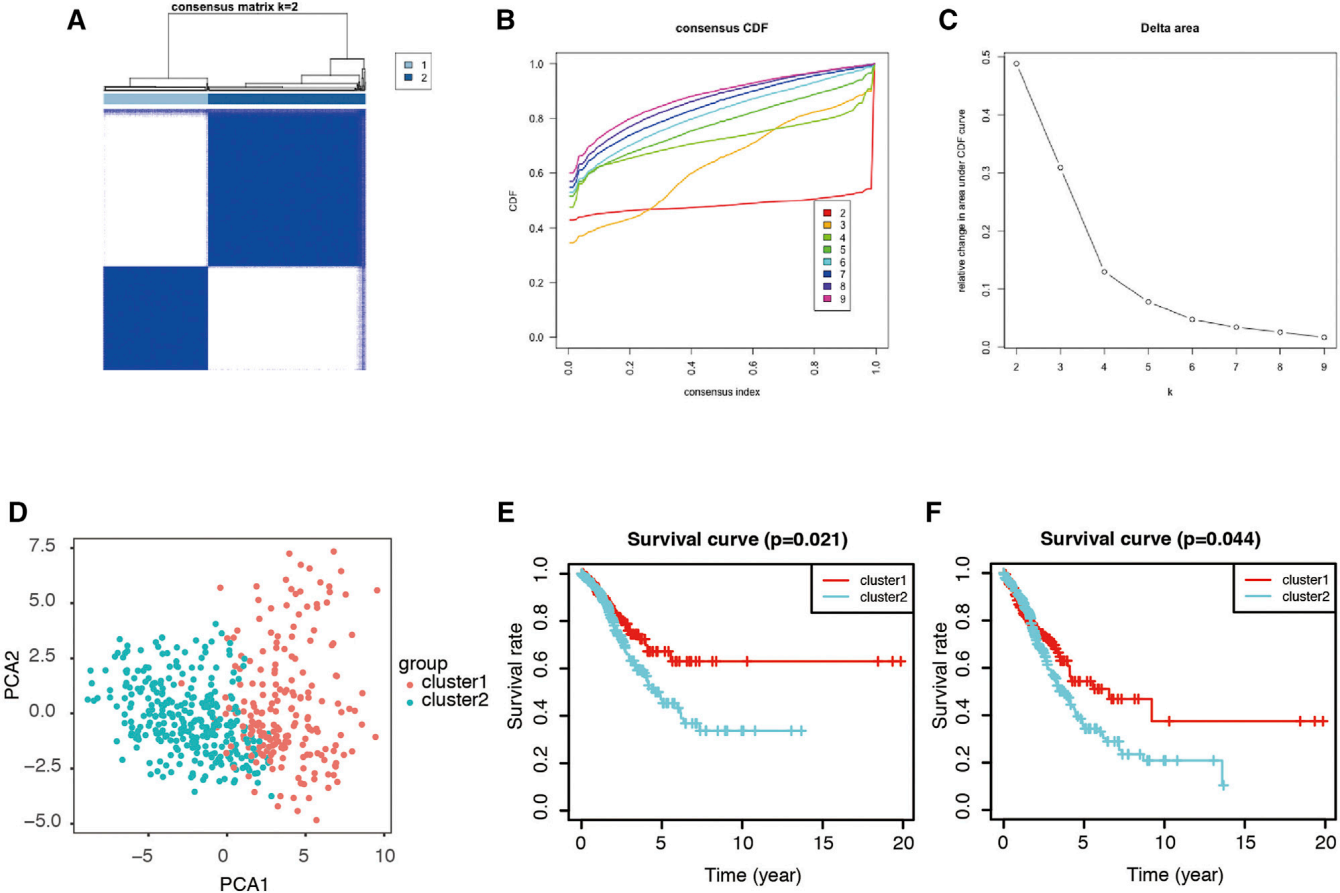
PCA analysis of genes in turquoise module. **(A)** Consensus matrix of two subgroups (k = 2). **(B,C)** Selection of k value. Consensus clustering cumulative distribution function (CDF) was set from k = 2 to 9. **(D)** Principal component analysis of the total RNA expression profile. **(E)** Kaplan-Meier overall survival (OS) curve for LUAD patients in cluster 1 and cluster 2. **(F)** Kaplan-Meier recurrence-free survival (RFS) curve for LUAD patients in cluster 1 and cluster 2.

ConcensusCluster analysis was utilized to classify the tumor samples. According to the expressional similarity of the gene expression in the turquoise module identified above as the powerful independent prognostic factors, k = 3 could be the optimal choice with clustering increasing from k = 2–9 (Figures 5A,B). However, we found that the interference found between subgroups only measured as minimal when k = 2. As such, we used k = 2 for consensus clustering analysis and, though this, we identified two subgroups named cluster 1 and cluster 2 (Figures 5C,D). Patients in cluster 1 were found to have a significantly shorter overall survival (OS, Figure 5E) and recurrence-free survival (RFS, Figure 5F) when compared with patients in cluster 2.

### Turquoise-Module-Based Prognosis Classifier and Clinicopathologic Characteristics in LUAD

**FIGURE 6:**
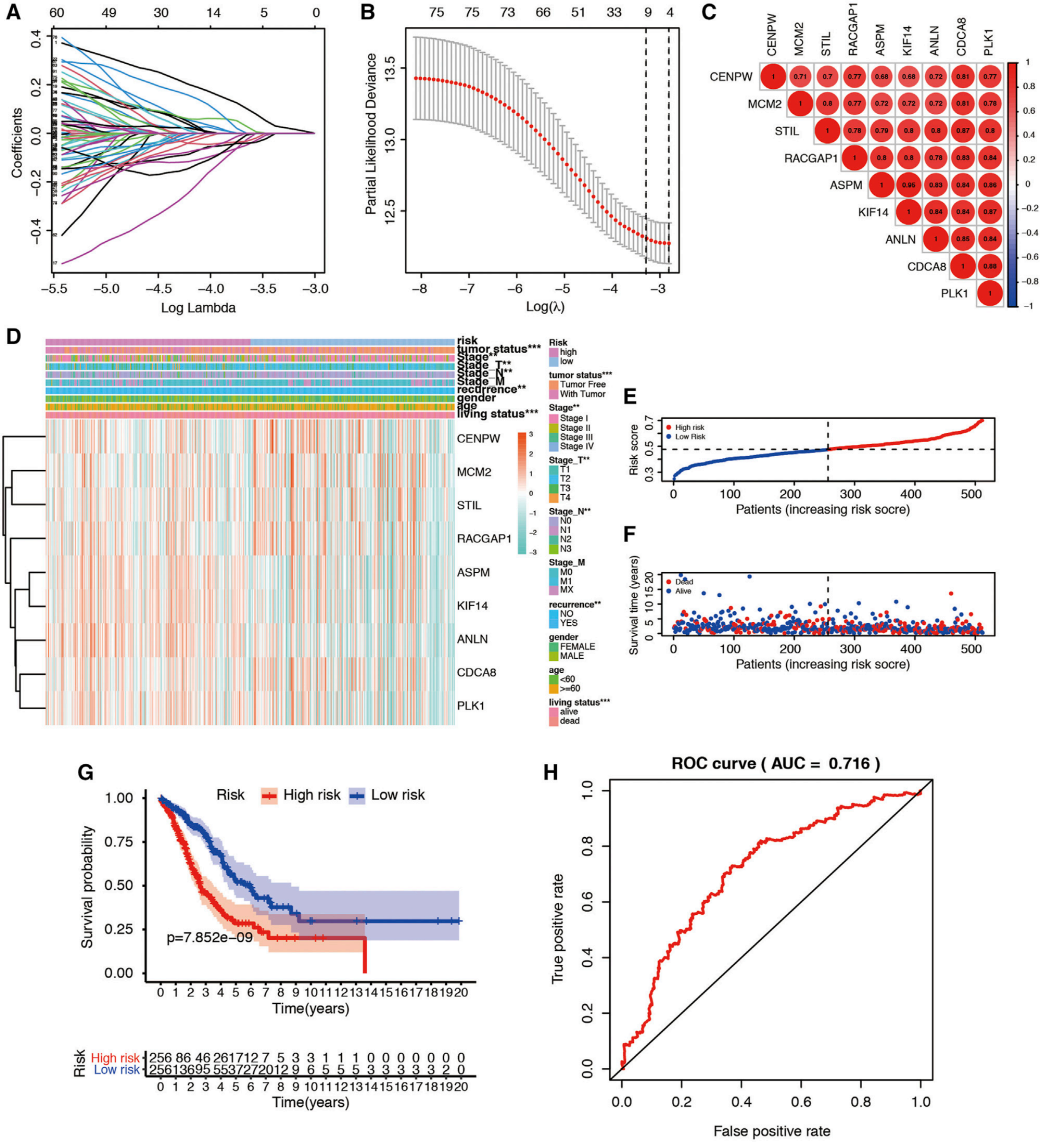
Construction and validation of mRNAsi-related signature for LUAD patients. **(A)** Lasso regression identified the prognostic model in LUAD. **(B)** Cross-validation to select the optimal tuning parameter (λ). The red dotted vertical line crosses over the optimal log λ. **(C)** The relationship among nine key genes. The bigger the circle size, the more correlative two genes are. **(D)** The heatmap shows the expression of the 9 genes in high-risk and low-risk LUAD patients. The distribution of clinicopathological characteristics was compared between the high-risk and low-risk groups. *p < 0.05, **p < 0.01 and ***p < 0.001. **(E)** Risk score distribution of patients in the prognostic model. **(F)** Survival status scatter plots for patients in the prognostic model. **(G)** Kaplan-Meier curve analysis of the high-risk and low-risk groups. **(H)** Time-dependent ROC curve analysis of the prognostic model.

To filter out an mRNAsi-based classifier to predict the prognosis of LUAD patients, a LASSO regression model was carried out using the 76 miRNAsi-related genes from the turquoise module. Furthermore, the nine mRNAsi survival-related DEG prognostic model (CENPW, MCM2, STIL, RACGAP1, ASPM, KIF14, ANLN, CDCA8, and PLK1) was constructed with LASSO regression to improve the predicted accuracy for overall survival in LUAD when log(lambda) was between -3.0 and −4.0 (Figures 6A,B). Risk scores were based on gene expression levels multiplied by its corresponding regression coefficient (Table 1). The formula was shown as: risk score = (ANLN*0.1574) (ASPM*0.0163) −(CDCA8*0.1315) +(CENPE*0.0241) +(KIF14*0.0598) −(MCM2*0.0431) +(PLK1*0.0907) −(RACGAP1*0.0916) −(STIL*0.0478). We then deeply analyzed the relationship among these 9 genes, and the results were shown in Figure 6C. Expression of the 9 genes and clinicopathological features in low- and high-risk patients from the TCGA dataset was demonstrated in the heatmap (Figure 6D). We found significant differences between the high- and low-risk groups associated with tumor status, stage_N, stage_T, AJCC stage, recurrence, and living status. We then used the median risk score to classify 513 LUAD samples as either low risk (n = 256) or high risk (n = 257) (Figures 6E,F). K-M survival curve indicated that patients in the high-risk group showed markedly poorer overall survival (OS) than those in the low-risk group (*p* = 7.852e−09; Figure 6G). The area under the ROC curve (AUC) for OS was 0.716 (Figure 6H), suggesting that this prognostic model exhibited a great sensitivity and specificity.

**TABLE 1:** Twelve hypoxia-associated genes and corresponding coefficient value.

Metabolic associated gene	Coefficient
ANLN	0.15745076
ASPM	0.01634707
CDCA8	−0.1315837
CENPE	0.0241496
KIF14	0.05981211
MCM2	−0.043114
PLK1	0.09071628
RACGAP1	−0.0916811
Risk score	Low: < 0.47
	High: ≥ 0.47

### Building Predictive Nomogram in LUAD Patients

**FIGURE 7:**
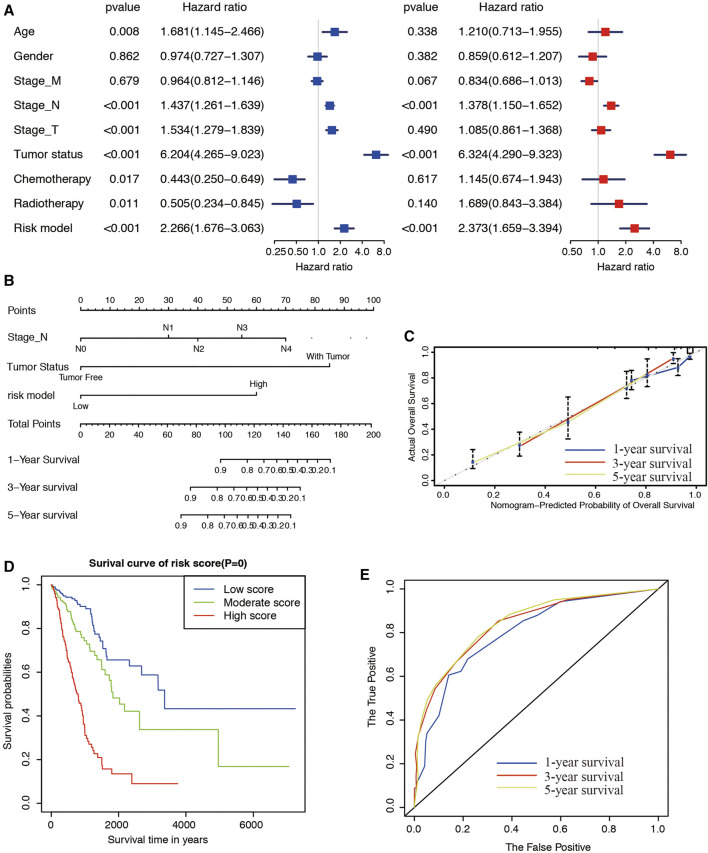
Construction of a nomogram for survival prediction. **(A)** Univariate and multivariate Cox analysis of clinical characteristics and the risk model. **(B)** A nomogram for predicting OS in patients with LUAD. **(C)** Calibration plot of 1-, 3- and 5-years actual risk probability was displayed, suggesting moderate potential for predicting survival for patients of LUAD. **(D)** Kaplan-Meier curve analysis of the low, moderate, and high score groups. **(E)** Comparison of time-dependent ROC curves predicting 1-, 3-, and 5-years overall survival.

To determine whether the risk signature could be used as an independent risk factor for LUAD patients, we performed univariate and multivariate Cox analyses (Figure 7A). The covariates included age, gender, T stages, M stages, N stages, chemotherapy, radiotherapy, tumor status, and risk model. Univariate Cox regression analysis showed that age, stage_T, stage_N, chemotherapy, radiotherapy, and risk model correlated with the prognosis of LUAD (*p* < 0.05). However, subsequent multivariate Cox analysis showed that the stage_N (HR = 1.378, 95% CI = 1.150–1.652, and *p* < 0.001), tumor status (HR = 6.324, 95% CI = 4.290–9.323, and *p* < 0.001), and risk model (HR = 2.373, 95% CI = 1.659–3.394, and *p* < 0.001) were independent risk factors for overall survival. We constructed nomogram maps to predict 1-, 3-, and 5-years overall survival in LUAD patients with stage_N, tumor status, and risk score (Figure 7B). The corresponding score of each factor was listed as Table 2. We validated the nomogram by calibration curve, and the 45° line represented the best prediction. Calibration plots indicated that the nomogram performed well in predicting 1-, 3-, and 5-years survival (Figure 7C). Patients were then divided into 3 subgroups, equally, according to the total score resulting from the nomogram. Kaplan-Meier survival curve revealed that patients in the low score subgroup had a better clinical outcome than those in the moderate and high score subgroups (Figure 7D). ROC curve analysis exhibited that the risk score AUC values of the model were 0.797, 0.821, and 0.836 for 1-, 3-, and 5-years survival, respectively (Figure 7E). These results reveal that the nomogram constructed by the 9-gene signature has a high accuracy in predicting the overall survival of LUAD patients.

**TABLE 2:** Corresponding risk score for each variable and total score.

Variables	Category	Score
Stage_N	N0	0
	N1	30
	N2	40
	N3	55
	N4	70
Tumor status	Tumor free	0
	With tumor	85
Risk model	Low	0
	High	60
Total score	Low risk	0–40
	Moderate risk	50–95
	High risk	≥100

### Validation of Risk Classifier in Two Independent Cohorts

**FIGURE 8:**
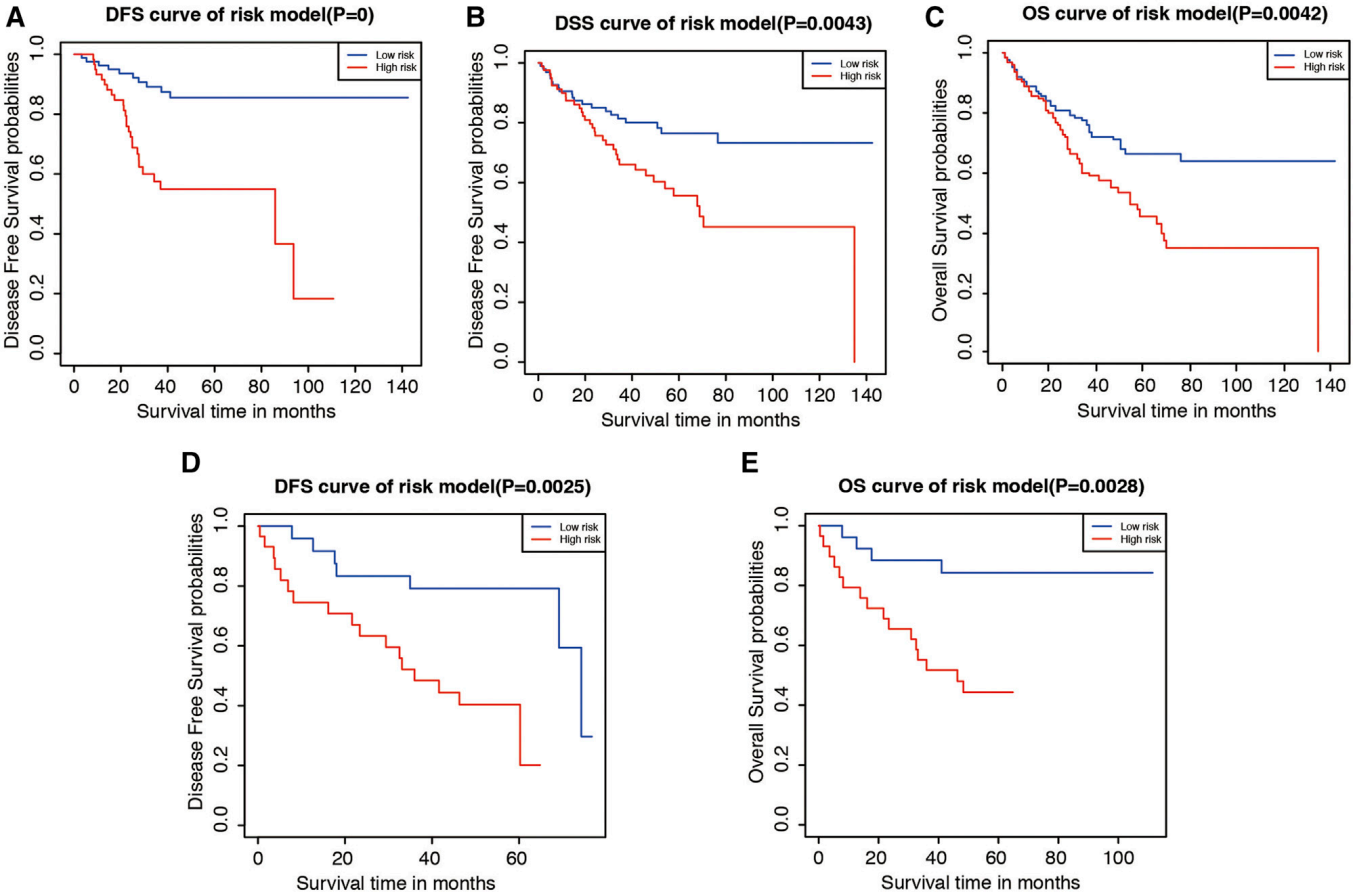
The validation of prognostic value of risk model in GSE17536 and GSE17537. **(A–C)**. Kaplan-Meier plot of disease-free survival (DFS), disease specific survival (DSS), and overall survival (OS) according to risk group for patients from GEO Series GSE17536. **(D,E)**. Kaplan-Meier plots of DSS and OS according to risk group for patients from GEO Series GSE17537.

The efficacy of the classifier was further evaluated using another outcome of different type of survivals, and the results were similar to what was seen in the TCGA cohort. To validate our signature, we first calculated the risk score for each patient according to the coefficient value of the 9 genes. Patients were divided into high-risk and low-risk groups with the median risk score utilized as the cut-off value. In the GSE17536 dataset, patients in the high-risk group had poorer outcomes in disease-free survival (DFS, Figure 8A), disease specific survival (DSS, Figure 8B), and overall survival (OS, Figure 8C). Similarly, in the GSE17537 dataset, patients in the high-risk group also had a worsened prognosis in DFS (Figure 8D) and OS (Figure 8E) when compared with those in the low-risk group. Altogether, this external validation indicated that risk signature is stable and highly precise in its prediction of LUAD patient prognosis.

## Discussion

NSCLC is a major cancer worldwide due to being associated with particularly high rates of mortality and morbidity. A major subtype of NSCLC, LUAD, and is not as well described as small cell lung cancer in terms of pathogenesis and risk factors (Herreros-Pomares et al., 2019). Poor outcomes is often associated with the development of drug resistance in lung cancer (Wu et al., 2020). However, a continually increasing number of studies have suggested an important role for cancer stem cells (CSCs). Therefore, it is urgent to do research on the therapeutic targets present in LUAD stem cells. A comprehensive study design, including an investigation of mRNAsi-related genes, may assist in the development of this innovative scientific perspective. In this study, we identified hub genes related to the mRNAsi in module by WGCNA with the TCGA database and the mRNAsi corresponding to each sample. Then genes from the turquoise module were selected and functional analysis of this module was conducted. We performed LASSO regression using genes from this module and built a robust 9-gene signature independent of clinical factors for predicting the OS of LUAD patients. Our results also indicated that these 9 genes are important factors in clinical characteristics and patients with high-risk scores were found to exhibit poor overall survival. External validation with GEO datasets also proved the stability and accuracy of this risk model. We constructed co-expression modules through WGCNA and the results indicated that three modules (blue, brown, and turquoise) had the greatest positive correlations with mRNAsi. The turquoise module was selected and key genes were screened from this module based on the GS and MM scores.

Numerous studies have revealed that cancer stem cells (CSCs) were thought to be a determinant of intratumor heterogeneity (Tang et al., 2021). CSCs have also been reported to be valuable in cancer research due to the important role they play in tumor differentiation, metastasis, drug resistance, and epigenetic alterations (La Noce et al., 2018). These theories led to Malta et al. suggesting a novel concept–the stemness index–to measure the development of tumors and evaluate how reliable stem cell indices are for assessing tumors using TCGA data (Malta et al., 2018). Some studies investigated the functional use of the stem cell indices in lung cancer, and found that several genes and pathways related to the immune system could help provide insight into potential associations between cancer stemness and the lung cancer microenvironment (Li et al., 2021). Another study identified a stem cell-related biomarker and provide a new strategy for exploring pathogenesis of LUAD (Zhao et al., 2020). However, they did not construct a prognostic risk signature and nomogram.

Next, GO analysis indicated that these genes were enriched in positive regulation of cell cycle, microtubule, ATP activity, and tubulin biding. It has been demonstrated that PMYT1 is important for G2/M arrest and may be a potential target for therapeutics (Long et al., 2020). Microtubule and tubulin binding are two critical components consisting of cytoskeleton. Cytoskeleton also plays an essential part in the progression of LUAD. A recent study concluded that deregulating Linc00426 reduced rearrangement of the cytoskeleton and matrix metalloproteinase expression, suggesting it may be a tumor marker for LUAD (Li et al., 2020). Pathway and function identified by KEGG included cell cycle and p53 signaling pathway. Previous studies have confirmed that these pathways are related to the occurrence, development, and drug resistance seen in LUAD patients (Huang et al., 2021; Zhu et al., 2021).

In this study, we identified 9 genes that we then used to construct our mRNAsi-related prognostic signature, and outcomes show that this prognostic tool had significant value in LUAD. The prediction performance of the above prognostic tool was determined to be satisfactory in LUAD patients by ROC. The risk signature, combined with clinicopathological features, resulted in high accuracy predictions of OS in LUAD patients, and AUC reached 0.836, which may help physicians develop more precise estimates of individual survival rates. Previous bioinformatics studies in LUAD have been conducted from a different angle. For example, one study’s prognostic signature was built using LASSO Cox regression that allowed them to predict the progression-free survival of LUAD patients, demonstrating that cancer stem cells may play an important role in the etiology of LUAD. (Liao et al., 2020). However, the accuracy of the risk model evaluated by AUC reached only 0.679. Another study established a prognostic predictive model for lung adenocarcinoma (LUAD) patients based on 13 metabolism-associated genes and validated the signature in external datasets. AUC at five year is 0.75 in the Okayama cohort (He et al., 2020). A detailed and comprehensive study of the co-occurring genetic abnormalities characterizing different LUAD subsets was also conducted for a better understanding of the disease heterogeneity, and for the discovery of new therapeutic targets (Testa et al., 2022).

Similarly in our study, in order to validate whether our risk model was efficient enough to predict the survival, we utilized other mRNA expression profiles, GSE17536 and GSE17537, from the GEO database as our testing set. The survival curves were plotted and the results proved that the 9-gene signature could significantly distinguish patients into low- and high-risk subgroups and the survival rates were diverse. MCM2 has been widely reported in lung cancer. The deregulation of MCM2 impacts lung cancer cell proliferation, the cell cycle, and cell migration. The mechanism revealed by multi-dimensional proteomic approaches might be conditioned via the regulation of HMGA1 phosphorylation (Cheung et al., 2017). The mitotic kinesin KIF14 has been previously shown to be overexpressed in a variety of cancers, including lung cancer. Corson et al. conducted an investigation into KIF14 expression and how it correlates with certain clinical variables, as well as how KIF14 alters *in vitro* colony formation in lung cancer. Their results indicated that expression of KIF14 is an independent prognostic variable for DFS in lung cancer and they found that knocking down KIF14 expression decreases tumorigenicity *in vitro*, suggesting that KIF14 is a potentially important marker in lung cancer that warrants further study (Corson et al., 2007). Other genes, including PLK1, CDCA8, ANLN, and RACGAP1, were all discovered to play different roles in proliferation, metastasis, and enhanced chemotherapy sensitivity to doxorubicin in lung cancer (De Carcer et al., 2018; Ge et al., 2019; Xu et al., 2019; Hu et al., 2021). Other significant biomarker such as BUB1B was found by a meta-analysis, and high BUB1B expression was associated with male sex, a smoking history, and an advanced TNM stage. High BUB1B expression was also a predictive of poor overall survival (OS) and progression-free survival (PFS) (Chen et al., 2021).

However, several limitations in this study must be acknowledged. First, the power and accuracy of the model could be improved by a large register-based series in our center. Second, the present study is purely computational from a public database, future experimental and clinical data are needed to validate the mechanism of the selected molecules. Finally, the data we used in this study originated from patient cohorts in the United States and are not representative of worldwide patient populations. Therefore, further studies utilizing larger and more diverse patient groups are needed to validate the findings within this study.

## Conclusion

In conclusion, we calculated and analyzed the mRNAsi of LUAD samples from the TCGA database based on their mRNA expression profiles. Our study revealed that the mRNAsi-related genes in the turquoise module are closely correlated with malignant clinicopathological characteristics of LUAD by WGCNA analysis. These genes could be classified into two clusters by PCA analysis and LASSO regression, and the results proved to be accurate in their classification of patients. External databases validated that the risk signature is highly accurate. In total, we provide a new strategy for exploring stemness-related genes in LUAD cases.

## Data Availability

The following information was supplied regarding data availability: the expression profile and clinical data are all from the level 3 data available in the TCGA database (https://portal.gdc.cancer.gov/repository). The microarray-based expression data of LADC patients and associated clinical information are available at Gene Expression Omnibus: GSE17536 and GSE17537.
